# Stepwise **MRI** mode activation in a patient with a non‐ **MRI** ‐conditional **VDD** lead

**DOI:** 10.1002/joa3.70089

**Published:** 2025-05-12

**Authors:** Risa Kanai, Yoshitaka Terazaki, Hitoshi Mori, Yoshifumi Ikeda, Ritsushi Kato

**Affiliations:** ^1^ Outpatient Clinic of Cardiology Saitama Medical University, International Medical Center Saitama Japan; ^2^ Department of Cardiology Saitama Medical University, International Medical Center Saitama Japan

**Keywords:** CIED, MRI, pacemaker

## Abstract

A Medtronic MRI‐conditional device connected to an MRI‐non‐conditional VDD lead, which required specific adjustments in MRI mode settings to enable safe imaging. Although switching to MRI mode from VDD mode or VVI mode was not possible, it became possible after switching to DDD mode.

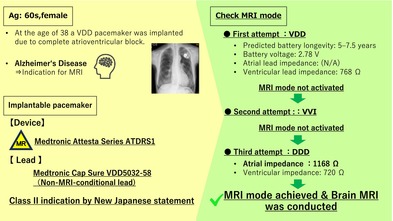

In Japan, a revised joint statement by three academic societies^1^ will take effect in January 2024, marking a significant change in MRI imaging protocols. Previously, MRI imaging was permitted only when MRI‐conditional leads were connected to MRI‐conditional devices with the same brand. The revision now allows imaging even when MRI‐conditional devices are connected to MRI‐conditional or non‐conditional leads with mixed brands. Here, we report a case involving a patient with an MRI‐conditional device connected to an MRI‐non‐conditional VDD lead, which required specific adjustments in MRI mode settings to enable safe imaging. This case highlights the importance of tailored device programming in such scenarios.

The patient is a woman in her 60s who underwent VDD pacemaker implantation at the age of 38 because of complete atrioventricular block. Subsequent replacement surgeries were performed at the ages of 46, 54, and 63 years. Following the most recent procedure, routine pacemaker checks were conducted at a nearby medical facility, with no notable complications reported. The patient, who had early‐onset Alzheimer's disease, required magnetic resonance imaging to assess eligibility for lecanemab therapy. However, MRI imaging could not be performed at a nearby medical facility, leading to a referral from a distant institution. Upon arrival, the patient's electrocardiogram showed atrial sensing and ventricular pacing at a rate of approximately 50 beats per minute (Figure 1). A pacemaker was implanted in the left anterior chest wall, with no abandoned leads present (Figure 2). The device in use was the Medtronic Attesta® Series ATDRS1, paired with a Medtronic Cap Sure® VDD5032‐58 lead, which is classified as MRI‐non‐conditional.

**FIGURE 1:**
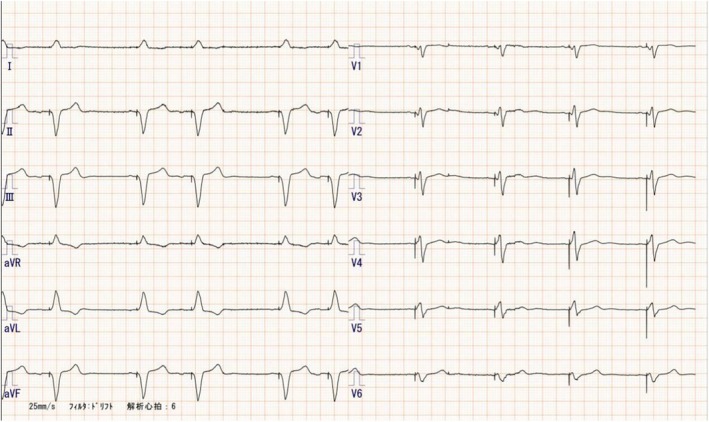
ECG. An ECG showed atrial sensing and ventricular pacing at a rate of approximately 50 beats per minute.

**FIGURE 2:**
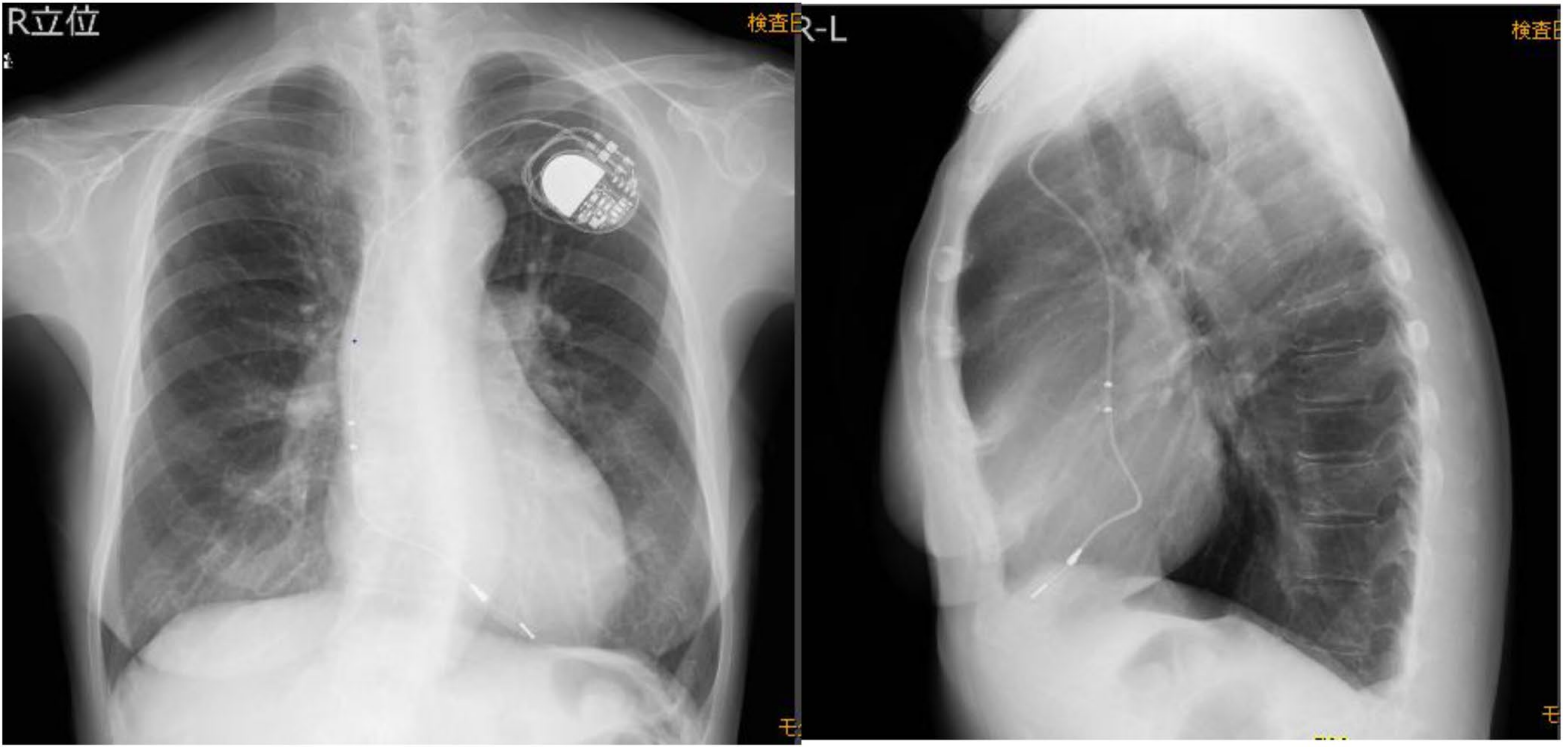
Chest x‐ray. A VDD pacemaker was implanted in the left anterior chest wall, with no abandoned leads.

Device diagnostics revealed the following:

- Predicted battery longevity: 5–7.5 years
- Battery voltage: 2.78 V
- Cell impedance: 358 Ω
- Ventricular lead impedance: 768 Ω
- Ventricular pacing threshold: 0.75 V at 0.4 ms
- Ventricular signal amplitude: Not detectable
- Atrial sensing amplitude: 0.7–1.0mv

For Class II imaging in revised Japanese statement, determining whether MRI mode can be activated is a critical consideration. Initial attempts to activate the MRI mode resulted in a programmer pop‐up message stating: *“MRI cannot be safely performed with this system. One or more lead impedances exceed the safety requirements or are unconfirmed.”* Subsequently, the pacemaker mode was switched to VVI, and another attempt to enable MRI mode was made, but the situation remained unchanged. Following this, the device was reprogrammed to DDD mode, allowing measurement of atrial impedance (1168 Ω). The mode was then changed to VOO, and another attempt to activate MRI mode was made, which proved successful (Figure 3). This stepwise reprogramming strategy underscores the importance of individualized device management in addressing MRI safety concerns for patients with MRI‐ non‐conditional leads, enabling the successful utilization of advanced imaging modalities.

**FIGURE 3:**
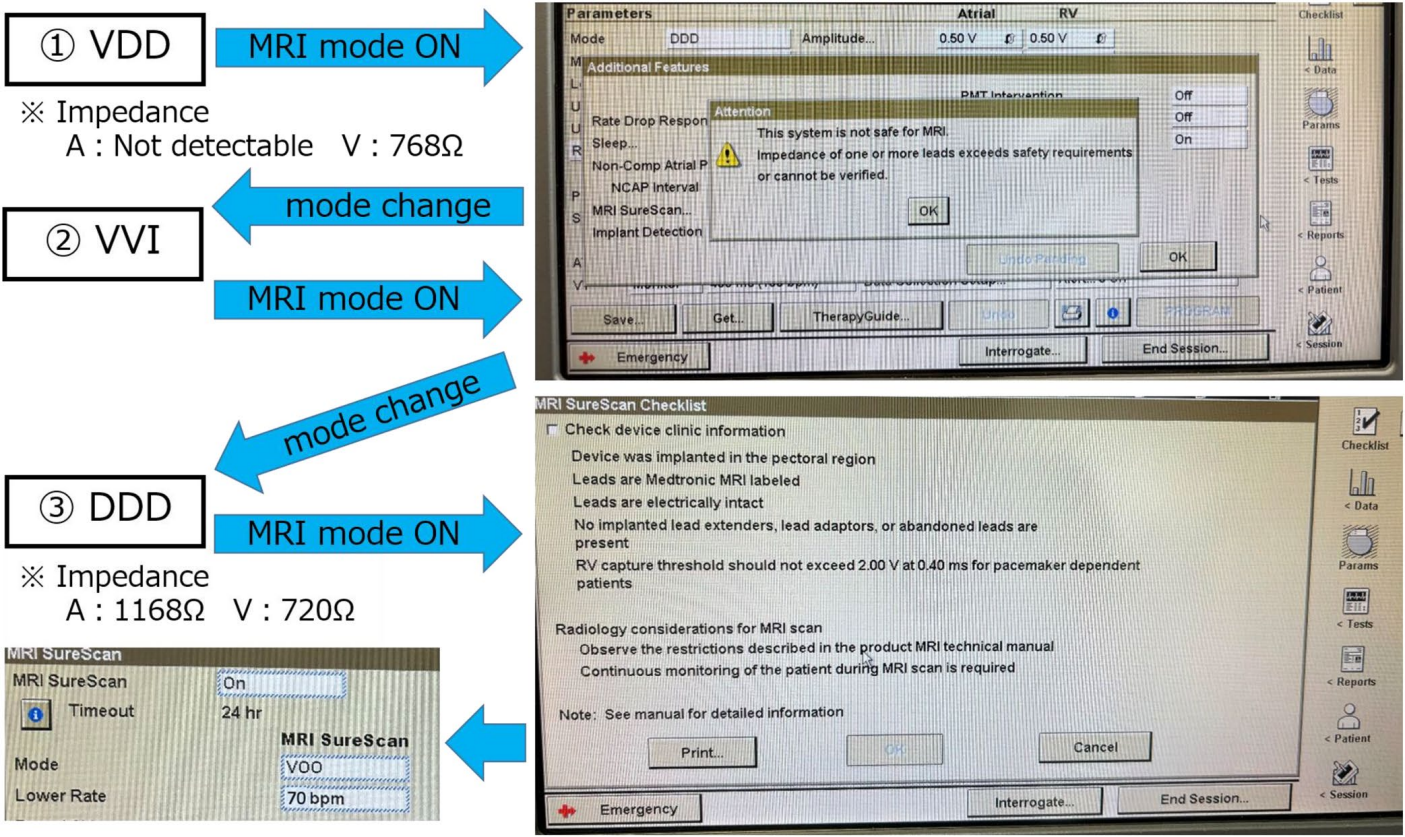
Steps to enter MRI Mode. Although switching to MRI mode from VDD mode or VVI mode was not possible, it became possible after switching to DDD mode.

Eleven days later, during the scheduled MRI imaging, the MRI mode was successfully activated. Given that the pacing rhythm was 100% atrial sensing and ventricular pacing mode, imaging was conducted in VOO mode at 70 bpm.

Post‐MRI device evaluation showed no complications, with the following parameters:

- Ventricular impedance: 722 Ω
- Ventricular pacing threshold: 0.5 V at 0.4 ms

The patient experienced no adverse events, and the MRI data were successfully delivered to the referring medical institution, enabling the initiation of lecanemab therapy.

## 1 DISCUSSION

In Japan, MRI‐conditional cardiac implantable electronic devices (CIEDs) have been reimbursed by insurance since 2012, enabling safe MRI examinations in medical institutions that meet specific facility standards.^1^ The safety of MRI conditional devices has been demonstrated in numerous studies. Overseas, many MRI scans have been safely performed even on non‐MRI‐conditional devices.^2^, ^3^ These findings have been reflected in the publication of guidelines concerning MRI examinations for patients with CIEDs. Notably, the Heart Rhythm Society in the United States issued guidelines in 2017, followed by the European Society of Cardiology in 2021.^4^, ^5^ These developments underscore the global effort to expand MRI accessibility for CIEDs patients while maintaining safety, emphasizing the importance of nuanced clinical approaches and evidence‐based practice in this evolving field. Reflecting that global perspective, a revised statement^1^ from the Japanese Society of Radiology, the Japanese Society for Magnetic Resonance in Medicine, and the Japanese Heart Rhythm Society will take effect on January 12, 2024. This revision has introduced a unique situation in Japan, where MRI imaging is possible only if the MRI mode can be configured for MRI conditional CIEDs, even if the leads are MRI non‐conditional leads. These scenarios emphasize the need for meticulous programming and individualized device assessment to ensure both patient safety and the efficacy of MRI examinations in this evolving regulatory framework.

In this case, the pacemaker was a Medtronic Attesta® Series ATDRS1 device connected to an MRI‐non‐conditional ventricular lead (Medtronic 5032–58). For MRI mode activation in Attesta® devices, two critical parameters must be verified: lead impedance and battery longevity. MRI mode activation is inhibited if any of the following conditions are met: (1) Impedance Issues: Lead impedance is below 200 Ω, exceeds 3000 Ω, or cannot be measured. (2) Battery Longevity: The device is at the Recommended Replacement Time (RRT), Elective Replacement Indicator (ERI), or End of Service (EOS) stage. These programmed restrictions ensure the safety of both the device and the patient during MRI imaging. The successful resolution of these challenges in this case required specific adjustments and careful monitoring of device parameters, demonstrating the importance of understanding these technical limitations in clinical practice. In this case, it was thought that measurement failure occurred because of VDD mode, as atrial leads are not measured during standard measurement. After switching to VVI mode, MRI mode could not be activated for the same reason. Therefore, the settings were changed to DDD, and after measuring the atrial lead impedance (which was within the acceptable range at 1168 Ω), the conditions for activating MRI mode were met. With pacemakers from Boston Scientific, Abbott, and Biotronik, it is possible to switch directly from VDD mode to MRI mode, and it is presumed that the phenomenon observed in this case would not occur. The requirement that atrial lead impedance must not be measured in VDD mode, and that the value must also fall within the normal range in order to switch to MRI mode, applies only to Medtronic and MicroPort devices. Therefore, caution is necessary when dealing with this phenomenon in pacemakers from these two manufacturers. Additionally, since devices will continue to evolve, it is important to constantly update our knowledge and adapt our approach to each individual case accordingly. There are likely to be various opinions regarding the acceptable range of impedance values. According to the latest statements, MRI is considered contraindicated for damaged leads. If the values fall within the range specified by the manufacturer, switching to MRI mode is possible. At our institution, MRI can be performed if a sufficiently safe margin in the pacing threshold can be ensured. However, if the margin is insufficient, we assess the risk–benefit balance and determine whether to proceed with MRI through a multidisciplinary conference.

In conclusion, this case highlights the importance of understanding and navigating device‐specific safety protocols to enable MRI examinations in complex clinical scenarios.

## CONFLICT OF INTEREST STATEMENT

The authors report no conflicts of interest.

## CONSENT

Informed consents were obtained from the patients to publish the case report.
